# Compressive Strength and Setting Time of MTA and Portland Cement Associated with Different Radiopacifying Agents

**DOI:** 10.5402/2012/898051

**Published:** 2012-08-23

**Authors:** Mario Tanomaru-Filho, Vanessa Morales, Guilherme F. da Silva, Roberta Bosso, José M. S. N. Reis, Marco A. H. Duarte, Juliane M. Guerreiro-Tanomaru

**Affiliations:** ^1^Department of Restorative Dentistry, Araraquara Dental School, São Paulo State University (UNESP), 14801-385 Araraquara, SP, Brazil; ^2^Department of Dentistry, Bauru Dental School, University of São Paulo (USP), 17012-901 Bauru, SP, Brazil

## Abstract

*Objective*. The aim of this study was to evaluate the compressive strength and setting time of MTA and Portland cement (PC) associated with bismuth oxide (BO), zirconium oxide (ZO), calcium tungstate (CT), and strontium carbonate (SC). *Methods*. For the compressive strength test, specimens were evaluated in an EMIC DL 2000 apparatus at 0.5 mm/min speed. For evaluation of setting time, each material was analyzed using Gilmore-type needles. The statistical analysis was performed with ANOVA and the Tukey tests, at 5% significance. *Results*. After 24 hours, the highest values were found for PC and PC + ZO. At 21 days, PC + BO showed the lowest compressive strength among all the groups. The initial setting time was greater for PC. The final setting time was greater for PC and PC + CT, and MTA had the lowest among the evaluated materials (*P* < 0.05). *Conclusion*. The results showed that all radiopacifying agents tested may potentially be used in association with PC to replace BO.

## 1. Introduction

Mineral Trioxide Aggregate (MTA) was introduced in the 1990s to seal communications between the periodontium and the root canal [1]. Nowadays, due to its good sealing ability [2] and biocompatibility [3], MTA is considered the ideal material of choice for retrograde fillings and treatment of root perforations.

Mineral Trioxide Aggregate is composed of Portland cement (PC) with the addition of bismuth oxide (BO) as a radiopacifying agent [4]. Since MTA is a Portland cement-based material, several studies have compared the physical, chemical, and biological properties of both cements [3, 4]. MTA and PC present antibacterial activity [5, 6] and biocompatibility: they donnot induce cell death or genotoxicity [7]. Moreover, this material promotes cellular growth and adhesion [8]. Additionally, PC is able to stimulate mineralized tissue formation [9] and, in rat subcutaneous tissue, it induces a mild inflammatory response, similarly to MTA [3]. 

Due to these characteristics, associated with low cost and availability, PC has been proposed as a clinical alternative material to MTA. Despite these favorable properties, PC does not present enough radiopacity to be distinguished from the adjacent anatomical structures, such as dentin and bone [10]. 

MTA contains 20% BO as a radiopacifying agent. BO confers good radiopacity both to commercially available MTA-based cements [11] and to PC [12]. However, this association has been questioned in relation to other physicochemical and biological properties. Bismuth oxide affected the hydration mechanism of MTA [13]. Besides, its presence increases the porosity of PC, which may lead to increased solubility and disintegration of the material, consequently affecting its resistance [14, 15]. BO has shown cytotoxicity towards dental pulp cells [8] and does not promote cell growth [16]. Consequently, the use of other radiopacifying agents with PC has been investigated [12, 17] in order to evaluate these mixtures in terms of their physicochemical and biological properties. 

Húngaro Duarte et al. [12] described that some substances such as zirconium oxide (ZO) and calcium tungstate (CT) added to the PC presented higher radiopacity than that of dentin and may potentially be used as radiopacifying agents in substitution to BO. Also, it was demonstrated that PC associated with ZO and CT is not cytotoxic in cell culture [18].

The aim of this study was to evaluate the compression strength and setting time of Portland cement associated with alternative radiopacifying agents.

## 2. Materials and Methods

The following experimental groups were established according to the materials to be tested: white MTA (Angelus, Londrina, PR, Brazil), PC (Irajazinho, Votorantim Cimentos, São Paulo, SP, Brazil), and PC with the addition of the following radiopacifying agents: BO (Sigma-Aldrich, St. Louis, MO, USA), zirconium oxide (ZO) (Sigma-Aldrich, St. Louis, MO, USA), calcium tungstate (CT) (Sigma-Aldrich, St. Louis, MO, USA), and strontium carbonate (SC) (Sigma-Aldrich, St. Louis, MO, USA). A ratio of 20% radiopacifying agent to 80% white PC, by mass, was used. Each material evaluated was mixed with 0.30 mL water for each 1 g of material [17].

### 2.1. Compression Strength

To test the compression strength, specimens measuring 12 mm in height by 6 mm in diameter were fabricated. The dimensions were used according to other cited references. Each experimental group included six specimens, which were maintained at 37°C under 100% relative humidity until the tests were performed. Each experimental group was subjected to testing at 24 hours and at 21 after-manipulation of the cements. The compression strength of each specimen was evaluated using a universal testing machine (EMIC DL 2000, Curitiba, PR, Brazil), at a speed of 0.5 mm/min and with a load of 5 kN. All measurements were recorded in kg and converted to megapascal (MPa).

### 2.2. Setting Time

This test was carried out as determined by the ADA specification 57 and ASTM specification C266-03. Six specimens measuring 10 mm in internal diameter and 2 mm in height were fabricated from each material. The initial and final setting times of the materials were determined using the Gilmore needles weighing 100 g and 456 g, respectively, according to the methodology described by Bortoluzzi et al. [19]. The initial and final setting times were determined by the arithmetic mean of six repetitions of the test for each experimental group. The mean setting times for the different cements were compared by ANOVA and the Tukey test (*P* < 0.05).

## 3. Results

The mean compression strength values of the different materials are shown in Table 1. PC, PC + ZO, and PC + SC had significantly higher compression strength values compared with the other experimental groups 24 hours after manipulation of the materials. At the same period, MTA and PC + BO had statistically lower mean compression strength values than the other groups. After 21 days, all the materials tested had similar compression strength values, except for PC + BO, which presented the lowest mean compression strength value (*P* < 0.05).

The mean initial and final setting times are shown in Table 2. PC had the highest initial setting time mean values compared to the other experimental groups (*P* < 0.05). Regarding final setting time, PC and PC + CT had significantly higher values compared to the other materials. On the other hand, MTA presented the lowest mean final setting time values among the materials tested.

## 4. Discussion

Mineral Trioxide Aggregate has been widely used in endodontics due to its good physicochemical properties and excellent biocompatibility [1, 3]. MTA and PC present similar compositions, except for the presence of BO, which is added to MTA as a radiopacifying agent [4].

The addition of 20% BO to PC promotes good radiopacity [11, 12] but may negatively affect the other properties of the cement [8, 14–16]. In this study, different radiopacifying agents were added to PC in order to allow comparison between the impact of these agents in specific properties like setting time and compression strength.

The obtained results demonstrated lower compression strength values for PC + BO compared with the other experimental materials, which is consistent with previous studies [14, 15]. BO does not participate in the hydration reaction of MTA [13]. Therefore, its presence may induce formation of flaws in the cement matrix, negatively affecting the mechanical strength of the product [14]. Furthermore, according to Coomaraswamy et al. [14], addition of BO to PC increases porosity by leaving more unreacted water within the set material. These flaws increase the solubility, the risk of fracture, and disintegration of the material, with marked decrease in its resistance [14]. 

Contrastingly, these observations differ from those reported by Saliba et al. [20], who verified that addition of BO to CP did not cause deterioration of the physical properties of the material. This difference in results may be due to several factors affecting the mechanical properties of the cement, such as the powder/liquid ratio, the size and shape of the particles, and different techniques of cement manipulation and of incorporating the powder into the liquid [17, 21]. 

Compressive strength tests demonstrated that except for the PC + BO group all the other experimental materials showed increase in their CS values from 24 hours to 21 days. According to Islam et al. [10], this is due to the continuous setting of the materials, which results in gaining strength and stability over time. 

ZO, CT, and SC did not affect the compressive strength of PC. ZO is an inert material widely used in orthopedic prostheses due to its biocompatibility, resistance to corrosion, and mechanical strength [22]. In dentistry, ZO has been used in prostheses and dental implants, presenting excellent biocompatibility and low toxicity [23]. Moreover, ZO, together with calcium tungstate, is the radiopacifying agent of AH Plus endodontic sealer, which presents outstanding radiopacity [24] and excellent biological properties, such as the ability to induce periapical repair [25]. Gomes Cornélio et al. [18] showed that PC associated with ZO and TC is not cytotoxic and may be good alternative as radiopacifying agent in substitution to BO. Húngaro Duarte et al. [12] observed that PC + ZO and PC + CT both had satisfactory radiopacity, lower than that presented by PC + BO, but still above the minimum values recommended by the ISO [26] and ADA [27] standards. 

As for the setting times, our results show higher initial and final setting times for PC compared with the other groups. This observation is in agreement with Camilleri [15], who reported significantly higher setting times for PC than for PC + BO. Nonetheless, these results contrast with previous studies in which the addition of radiopacifying agents increased the setting times of the materials [10, 17]. According to Neville [21], addition of any substance can interfere with the hydration mechanism of PC, delaying matrix formation, and consequently increasing the setting time of the cement. In the present study, setting times of MTA were lower than those of PC, which contrasts with results reported by some earlier studies [10, 17]. However, these differences may be related by the type of MTA used in the present study (MTA-Angelus), which is known to present lower setting times in comparison with ProRoot MTA (Dentsply, Maillefer, Switzerland). The mean setting time values found in the present study are similar to those reported by Bortoluzzi et al. [19], who also used MTA-Angelus.

## 5. Conclusion

The obtained results suggest that all radiopacifying agents tested may potentially be used as alternatives to BO in the formulation of MTA-based materials. Complementary studies are necessary to evaluate the behavior of these materials associated to PC regarding other physicochemical and biological properties before clinical recommendations can be done.

## Figures and Tables

**Table 1:** Means and standard deviations (SD) for compressive strength values (in MPa) at 24 hours and at 21 days after manipulation of each cement evaluated.

Cement	24 hours (MPa)	SD	21 days (MPa)	SD
PC	37.1^a^	4	41.2^a^	3.4
MTA	14.3^c^	3	43.4^a^	6.5
PC + BO	15.4^c^	1.6	22.9^c^	4.8
PC + ZO	38.5^a^	7.4	37.1^a^	7.4
PC + CT	26.6^b^	4	36.6^a^	8.3
PC + SC	38.2^a^	3.3	32.6^b^	3.9

PC: Portland cement; MTA: white MTA-Angelus; BO: bismuth oxide; ZO: zirconium oxide; CT: calcium tungstate; SC: strontium carbonate.

Different superscript letters (a, b, and c) indicate a statistically significant difference (*P* < 0.05).

**Table 2:** Means and standard deviations (SD) for the initial and final setting times (in minutes) for each cement evaluated.

Cement	Initial (min)	SD	Final (min)	SD
PC	25.5^a^	1.6	84.1^a^	8.6
MTA	13.5^b^	1.2	48.3^c^	4.0
PC + BO	13.7^b^	0.8	66.7^b^	3.5
PC + ZO	15.5^b^	1.2	73.0^b^	4.0
PC + CT	14.6^b^	0.6	80.5^a^	5.7
PC + SC	12.2^b^	1.4	73.1^b^	7.3

PC: Portland cement; MTA: white MTA-Angelus; BO: bismuth oxide; ZO: zirconium oxide; CT: calcium tungstate; SC: strontium carbonate.

Different superscript letters (a, b, and c) indicate a statistically significant difference (*P* < 0.05).
